# Aqua­bromidobis(dimethyl­glyoximato)cobalt(III)

**DOI:** 10.1107/S1600536811008877

**Published:** 2011-03-15

**Authors:** Parthasarathy Meera, Madhavan Amutha Selvi, Pachaimuthu Jothi, Arunachalam Dayalan

**Affiliations:** a Loyola College (Autonomous), Chennai 600 034, Tamil Nadu, India

## Abstract

In the title complex, [CoBr(C_4_H_7_N_2_O_2_)_2_(H_2_O)], a crystallo­graphic mirror plane bis­ects the mol­ecule, perpendicular to the glyoximate ligands. The geometry around the cobalt(III) atom is approximately octa­hedral with the four glyoximate N atoms forming the square base. A bromide ion and the O atom of a water mol­ecule occupy the remaining coordination sites. The N—Co—N bite angles are 82.18 (4) and 80.03 (16)°. The glyoximate moieties form strong intra­molecular O—H⋯O hydrogen bonds. The coordinated water mol­ecule forms an inter­molecular O—H⋯O hydrogen bond with a glyoximate O atom, thereby generating supra­molecular chains parallel to [010].

## Related literature

For related complexes, see: Ohkubo & Fukuzumi (2005 ▶); Randall & Alberty (1970 ▶); Schrauzer (1968 ▶); Trommel *et al.* (2001 ▶). For similar structures, see: Bernstein *et al.* (1995 ▶); Mégnamisi-Bélombé *et al.* (1983 ▶); Meera *et al.* (2009 ▶); Ramesh *et al.* (2008 ▶). For the preparation of similar complexes, see: Vijayraghavan & Dayalan (1992 ▶). For spectroscopic studies related to the title complex, see: Folgando *et al.* (1986 ▶); Khan *et al.* (1997 ▶); Lopez *et al.* (1986 ▶).
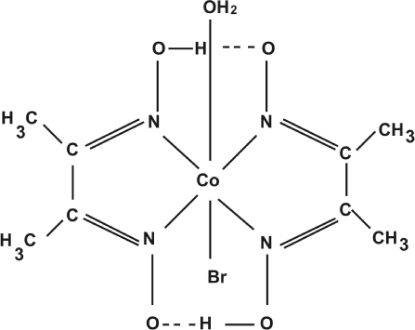


## Experimental

### 

#### Crystal data

[CoBr(C_4_H_7_N_2_O_2_)_2_(H_2_O)]
*M* *_r_* = 387.09
Monoclinic, 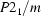

*a* = 7.5903 (3) Å
*b* = 8.8816 (4) Å
*c* = 10.5343 (5) Å
β = 96.137 (3)°
*V* = 706.09 (5) Å^3^
*Z* = 2
Mo *K*α radiation
μ = 4.07 mm^−1^
*T* = 293 K
0.15 × 0.10 × 0.10 mm

#### Data collection

Bruker Kappa APEXII CCD diffractometer
Absorption correction: multi-scan (*SADABS*; Bruker 1999 ▶) *T*_min_ = 0.581, *T*_max_ = 0.687
7395 measured reflections
1480 independent reflections
1298 reflections with *I* > 2σ(*I*)
*R*_int_ = 0.028

#### Refinement

*R*[*F* ^2^ > 2σ(*F* ^2^)] = 0.034
*wR*(*F* ^2^) = 0.096
*S* = 1.22
1480 reflections
101 parameters
2 restraints
H atoms treated by a mixture of independent and constrained refinement
Δρ_max_ = 1.01 e Å^−3^
Δρ_min_ = −0.54 e Å^−3^

### 

Data collection: *APEX2* (Bruker, 2004 ▶); cell refinement: *APEX2* and *SAINT-Plus* (Bruker, 2004 ▶); data reduction: *SAINT-Plus* and *XPREP* (Bruker, 2004 ▶); program(s) used to solve structure: *SIR92* (Altomare *et al.*, 1993 ▶); program(s) used to refine structure: *SHELXL97* (Sheldrick, 2008 ▶); molecular graphics: *ORTEP-3* (Farrugia, 1997 ▶) and *Mercury* (Macrae *et al.*, 2008) ▶; software used to prepare material for publication: *PLATON* (Spek, 2009 ▶).

## Supplementary Material

Crystal structure: contains datablocks global, I. DOI: 10.1107/S1600536811008877/fj2399sup1.cif

Structure factors: contains datablocks I. DOI: 10.1107/S1600536811008877/fj2399Isup2.hkl

Additional supplementary materials:  crystallographic information; 3D view; checkCIF report

## Figures and Tables

**Table 1:** Hydrogen-bond geometry (Å, °)

*D*—H⋯*A*	*D*—H	H⋯*A*	*D*⋯*A*	*D*—H⋯*A*
O2—H2⋯O1^i^	0.92 (1)	1.58 (1)	2.494 (3)	169 (4)
O3—H3⋯O1^ii^	0.85 (3)	1.79 (3)	2.616 (3)	167 (4)

Symmetry codes: (i) 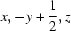; (ii) 
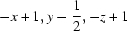
.

**Fig. 1.**
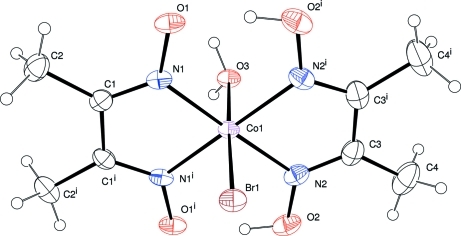
Displacement ellipsoid plot of the title compound drawn at 30% probability level.The equivalent symbol i represents the mirror symmetry (x, 1/2; -y,z) at one fourth of b axes.

**Fig. 2.**
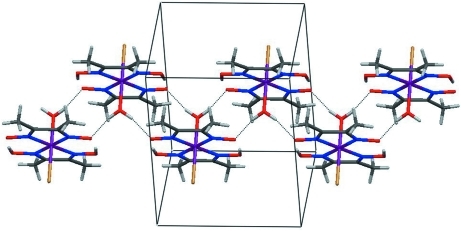
Part of the crystal structure of the title compound showing the formation of one dimensional chain through O3—H3···O1ii hydrogen bond extending along [010] direction [Symmetry codes: (i) x, -y + 1/2, z; (ii) -x + 1, y - 1/2, -z + 1].

## supplementary crystallographic information

### Comment

A number of cobalt complexes have been proposed as model systems for vitamin-B_12_(Trommel *et al.*, 2001; Ohkubo & Fukuzumi, 2005). The most commonly mentioned model system is bis(dimethylglyoximato)cobalt(III) complexes on which Schrauzer has carried out a great amount of research.The common feature of the different models is that each possesses a strong equatorial ligand field (Schrauzer,1968). A variety of cobalt(III) complexes have been discovered possessing stable axial cobalt-carbon bonds. Simple alkyl cobaloximes, are thermally stable upto about 200°C and are therefore among the most stable organo metallic compounds known. Halide ions can coordinate to cobalt(III) as other common anionic ligands. Cobalt(III) complexes, being low spin, are conveniently studied in aqueous medium (Randall & Alberty, 1970). We report here the synthesis and X-ray crystal structure of the title compound.

The geometry around the cobalt(III) is approximately octahedral with the four glyoximate N atoms forming the square base;whereas, the coordinated bromide (Br1) and oxygen (O3) and the coordinated oxygen of water form the apex. The bite angles of the glyoximates with cobalt are N(1)#1-Co(1)—N(1) 82.18 (14)° and N(2)#1-Co(1)—N(2) 80.03 (03)°, respectively. Further N(1)#1-Co(1)—N(2)#1 178.29 (10)° confirms the distorted octahedral geometry of the molecule. The bond lengths Co(1)—N(1)#1, 1.883 (2) Å,Co(1)—N(2)#1, 1.911 (2) Å agree well with the previously reported structures (Meera *et al.*, 2009, Ramesh *et al.*, 2008) and the axial Co–Br distance d(Co1–Br1) = 2.3563 (6)Å agrees well with the reported structure of *trans*-aquabromobis[ethanedial dioximato(1-)-N,*N*']cobalt(III)(Mégnamisi-Bélombé *et al.*, 1983). The glyoximate moieties are further bound by strong intraomecular O—H···O hydrogen bonds showing an *S*(6) ring motif (Bernstein *et al.*, 1995). Thecoordinated water forms an intermolecular hydrogen bond O3—H3···O1^ii^[symmetry code (ii): -*x* + 1, *y* - 1/2, -*z* + 1] with the glyoximate oxygen atoms which links the inversion related title compound thus forming a ring motif of R_2_^2^(10). Fused rings of R_2_^2^(10) generates a supramolecular one dimensional chain extending parallel to [010] direction. The structure is further stabilized through van der waals interaction.

### Experimental

Cobalt(II) bromide hexahydrate was thoroughly grinded and exposed to microwave for 30 s.The dehydrated cobalt(II) bromide was mixed with dimethylglyoxime in 1:2 molar ratio in acetone medium and allowed to stir for an hour (Vijayraghavan & Dayalan, 1992). The dibromo complex obtained was filtered dried and then it was refluxed with water for two hours. The resulting brown mass was filtered washed with ether and dried over desiccator. The elemental analysis data, obtained by analytical methods agree well with the theoretical data expected for the formula of the complex, C_8_H_16_N_4_O_5_BrCo proposed *viz*.,[Co(dmgH)_2_(H_2_O)Br]: Anal,% (cald,%): C, 25.12(24.8); H,4.82(4.13); N,14.50(14.47). The C=N stretching vibration of oxime in its complex was observed at 1580 cm^-1^ and the intra molecular hydrogen bonded OH around 3100 cm^-1^. A moderate peak around 1070 cm^-1^ may be assigned to the C=N—O stretching of the oxime. The peak around 510 cm^-1^ could be attributed to cobalt(III)-nitrogen stretching (Khan *et al.*, 1997; Folgando *et al.*, 1986). The ^1^H NMR spectra of the complex in DMSO-d_6_ shows a sharp intense singlet at 2.3 p.p.m. corresponding to methyl protons of the oxime.The oxime –OH resonates at 13.08 p.p.m..A singlet around 8.5 ppm represents the –OH of the aquo ligand (Lopez *et al.*, 1986).

### Refinement

The H– atoms bound to C– atoms were constrained to riding atoms with d(C—H) = 0.96Å and *U*_iso_(H) = 1.5*U*_equ_(C).The positions of the hydrogen atoms,bound to the glyoximate and water O atoms, were identified from difference in the electron density map and restrained to a distance of d(O2—H2) = 0.92 (1)Å and d(O3—H3) = 0.85 (1) Å. A difference elctron density peak of 1.008 e A^-3^ was observed after the final refinement. Since the observed peak position is meaningless it is ignored.

### Crystal data

**Table:**

[CoBr(C_4_H_7_N_2_O_2_)_2_(H_2_O)]	*F*(000) = 388
*M**_r_* = 387.09	*D*_x_ = 1.821 Mg m^−^^3^
Monoclinic, *P*2_1_/*m*	Mo *K*α radiation, λ = 0.71073 Å
Hall symbol: -P 2yb	Cell parameters from 3745 reflections
*a* = 7.5903 (3) Å	θ = 2.7–30.7°
*b* = 8.8816 (4) Å	µ = 4.07 mm^−^^1^
*c* = 10.5343 (5) Å	*T* = 293 K
β = 96.137 (3)°	Block, brown
*V* = 706.09 (5) Å^3^	0.15 × 0.10 × 0.10 mm
*Z* = 2	

### Data collection

**Table:**

Bruker Kappa APEXII CCD diffractometer	1480 independent reflections
Radiation source: fine-focus sealed tube	1298 reflections with *I* > 2σ(*I*)
graphite	*R*_int_ = 0.028
ω and φ scans	θ_max_ = 26.0°, θ_min_ = 2.7°
Absorption correction: multi-scan (*SADABS*; Bruker 1999)	*h* = −9→9
*T*_min_ = 0.581, *T*_max_ = 0.687	*k* = −10→9
7395 measured reflections	*l* = −12→12

### Refinement

**Table:**

Refinement on *F*^2^	Primary atom site location: structure-invariant direct methods
Least-squares matrix: full	Secondary atom site location: difference Fourier map
*R*[*F*^2^ > 2σ(*F*^2^)] = 0.034	Hydrogen site location: inferred from neighbouring sites
*wR*(*F*^2^) = 0.096	H atoms treated by a mixture of independent and constrained refinement
*S* = 1.22	*w* = 1/[σ^2^(*F*_o_^2^) + (0.0564*P*)^2^ + 0.1583*P*] where *P* = (*F*_o_^2^ + 2*F*_c_^2^)/3
1480 reflections	(Δ/σ)_max_ = 0.001
101 parameters	Δρ_max_ = 1.01 e Å^−^^3^
2 restraints	Δρ_min_ = −0.54 e Å^−^^3^

### Special details

**Table:**

Geometry. All e.s.d.'s (except the e.s.d. in the dihedral angle between two l.s. planes) are estimated using the full covariance matrix. The cell e.s.d.'s are taken into account individually in the estimation of e.s.d.'s in distances, angles and torsion angles; correlations between e.s.d.'s in cell parameters are only used when they are defined by crystal symmetry. An approximate (isotropic) treatment of cell e.s.d.'s is used for estimating e.s.d.'s involving l.s. planes.
Refinement. Refinement of *F*^2^ against ALL reflections. The weighted *R*-factor *wR* and goodness of fit *S* are based on *F*^2^, conventional *R*-factors *R* are based on *F*, with *F* set to zero for negative *F*^2^. The threshold expression of *F*^2^ > σ(*F*^2^) is used only for calculating *R*-factors(gt) *etc*. and is not relevant to the choice of reflections for refinement. *R*-factors based on *F*^2^ are statistically about twice as large as those based on *F*, and *R*- factors based on ALL data will be even larger.

### Fractional atomic coordinates and isotropic or equivalent isotropic displacement parameters (Å^2^)

**Table:**

	*x*	*y*	*z*	*U*_iso_*/*U*_eq_	
C1	0.7937 (4)	0.3331 (3)	0.5395 (3)	0.0330 (6)	
C2	0.8935 (4)	0.4238 (4)	0.6415 (3)	0.0487 (8)	
H2A	0.8818	0.5288	0.6207	0.073*	
H2B	1.0164	0.3960	0.6486	0.073*	
H2C	0.8469	0.4052	0.7213	0.073*	
C3	0.3600 (4)	0.1669 (4)	0.1163 (3)	0.0448 (8)	
C4	0.2476 (6)	0.0761 (5)	0.0221 (4)	0.0726 (13)	
H4A	0.2737	−0.0288	0.0358	0.109*	
H4B	0.2709	0.1038	−0.0626	0.109*	
H4C	0.1250	0.0942	0.0318	0.109*	
N1	0.6996 (3)	0.3893 (2)	0.4417 (2)	0.0302 (5)	
N2	0.4668 (3)	0.1117 (3)	0.2069 (2)	0.0374 (6)	
O1	0.6810 (3)	0.5377 (2)	0.4230 (2)	0.0395 (5)	
O2	0.4819 (3)	−0.0402 (3)	0.2186 (2)	0.0501 (6)	
O3	0.3771 (4)	0.2500	0.4145 (3)	0.0314 (6)	
Co1	0.58731 (6)	0.2500	0.32505 (5)	0.02601 (18)	
Br1	0.83644 (6)	0.2500	0.20942 (4)	0.04048 (18)	
H2	0.557 (4)	−0.051 (4)	0.293 (2)	0.059 (12)*	
H3	0.366 (5)	0.173 (3)	0.460 (3)	0.050 (10)*	

### Atomic displacement parameters (Å^2^)

**Table:**

	*U*^11^	*U*^22^	*U*^33^	*U*^12^	*U*^13^	*U*^23^
C1	0.0234 (13)	0.0376 (16)	0.0391 (15)	−0.0028 (12)	0.0091 (11)	−0.0058 (12)
C2	0.0354 (17)	0.060 (2)	0.0503 (18)	−0.0080 (15)	0.0039 (14)	−0.0164 (17)
C3	0.0321 (15)	0.068 (2)	0.0356 (16)	−0.0038 (15)	0.0097 (13)	−0.0091 (15)
C4	0.054 (2)	0.106 (4)	0.056 (2)	−0.018 (2)	0.0022 (19)	−0.030 (2)
N1	0.0284 (12)	0.0230 (12)	0.0415 (13)	−0.0032 (9)	0.0139 (10)	−0.0029 (10)
N2	0.0327 (13)	0.0388 (15)	0.0430 (14)	−0.0053 (11)	0.0140 (11)	−0.0082 (11)
O1	0.0446 (12)	0.0229 (10)	0.0538 (13)	−0.0022 (9)	0.0180 (10)	−0.0031 (9)
O2	0.0540 (15)	0.0376 (13)	0.0604 (15)	−0.0075 (11)	0.0142 (12)	−0.0163 (11)
O3	0.0300 (14)	0.0259 (15)	0.0401 (16)	0.000	0.0130 (12)	0.000
Co1	0.0251 (3)	0.0227 (3)	0.0312 (3)	0.000	0.0076 (2)	0.000
Br1	0.0364 (3)	0.0432 (3)	0.0441 (3)	0.000	0.01498 (19)	0.000

### Geometric parameters (Å, °)

**Table:**

C1—N1	1.289 (4)	C4—H4C	0.9600
C1—C1^i^	1.476 (6)	N1—O1	1.338 (3)
C1—C2	1.485 (4)	N1—Co1	1.883 (2)
C2—H2A	0.9600	N2—O2	1.358 (3)
C2—H2B	0.9600	N2—Co1	1.911 (2)
C2—H2C	0.9600	O2—H2	0.921 (10)
C3—N2	1.283 (4)	O3—Co1	1.938 (3)
C3—C3^i^	1.475 (7)	O3—H3	0.85 (3)
C3—C4	1.477 (4)	Co1—N1^i^	1.883 (2)
C4—H4A	0.9600	Co1—N2^i^	1.911 (2)
C4—H4B	0.9600	Co1—Br1	2.3563 (6)
			
N1—C1—C1^i^	112.79 (16)	C3—N2—O2	119.2 (3)
N1—C1—C2	124.4 (3)	C3—N2—Co1	117.3 (2)
C1^i^—C1—C2	122.85 (19)	O2—N2—Co1	123.3 (2)
C1—C2—H2A	109.5	N2—O2—H2	103 (2)
C1—C2—H2B	109.5	Co1—O3—H3	114 (3)
H2A—C2—H2B	109.5	N1—Co1—N1^i^	82.18 (14)
C1—C2—H2C	109.5	N1—Co1—N2^i^	98.88 (11)
H2A—C2—H2C	109.5	N1^i^—Co1—N2^i^	178.29 (10)
H2B—C2—H2C	109.5	N1—Co1—N2	178.29 (10)
N2—C3—C3^i^	112.51 (19)	N1^i^—Co1—N2	98.88 (11)
N2—C3—C4	124.4 (4)	N2^i^—Co1—N2	80.03 (16)
C3^i^—C3—C4	123.1 (2)	N1—Co1—O3	91.24 (9)
C3—C4—H4A	109.5	N1^i^—Co1—O3	91.24 (9)
C3—C4—H4B	109.5	N2^i^—Co1—O3	87.40 (10)
H4A—C4—H4B	109.5	N2—Co1—O3	87.40 (10)
C3—C4—H4C	109.5	N1—Co1—Br1	90.29 (7)
H4A—C4—H4C	109.5	N1^i^—Co1—Br1	90.29 (7)
H4B—C4—H4C	109.5	N2^i^—Co1—Br1	91.04 (7)
C1—N1—O1	122.7 (2)	N2—Co1—Br1	91.04 (7)
C1—N1—Co1	116.1 (2)	O3—Co1—Br1	177.97 (9)
O1—N1—Co1	121.18 (18)		
			
C1^i^—C1—N1—O1	179.81 (18)	C1—N1—Co1—O3	91.1 (2)
C2—C1—N1—O1	−0.7 (4)	O1—N1—Co1—O3	−88.7 (2)
C1^i^—C1—N1—Co1	0.02 (19)	C1—N1—Co1—Br1	−90.28 (19)
C2—C1—N1—Co1	179.5 (2)	O1—N1—Co1—Br1	89.92 (19)
C3^i^—C3—N2—O2	−179.62 (19)	C3—N2—Co1—N1^i^	174.1 (2)
C4—C3—N2—O2	0.8 (5)	O2—N2—Co1—N1^i^	−2.4 (2)
C3^i^—C3—N2—Co1	3.8 (2)	C3—N2—Co1—N2^i^	−4.6 (3)
C4—C3—N2—Co1	−175.8 (3)	O2—N2—Co1—N2^i^	178.98 (17)
C1—N1—Co1—N1^i^	0.0 (2)	C3—N2—Co1—O3	83.2 (2)
O1—N1—Co1—N1^i^	−179.82 (14)	O2—N2—Co1—O3	−93.2 (2)
C1—N1—Co1—N2^i^	178.6 (2)	C3—N2—Co1—Br1	−95.5 (2)
O1—N1—Co1—N2^i^	−1.2 (2)	O2—N2—Co1—Br1	88.1 (2)

Symmetry codes: (i) *x*, −*y*+1/2, *z*.

### Hydrogen-bond geometry (Å, °)

**Table:**

*D*—H···*A*	*D*—H	H···*A*	*D*···*A*	*D*—H···*A*
O2—H2···O1^i^	0.92 (1)	1.58 (1)	2.494 (3)	169 (4)
O3—H3···O1^ii^	0.85 (3)	1.79 (3)	2.616 (3)	167 (4)

Symmetry codes: (i) *x*, −*y*+1/2, *z*; (ii) −*x*+1, *y*−1/2, −*z*+1.
